# CHILDREN’S HEALTH: AAP Publishes Oil Protection Guidelines for Children

**DOI:** 10.1289/ehp.118-a431

**Published:** 2010-10

**Authors:** Bob Weinhold

**Affiliations:** **Bob Weinhold**, MA, has covered environmental health issues for numerous outlets since 1996. He is a member of the Society of Environmental Journalists

Sparked by requests from anxious parents throughout the U.S. Gulf Coast region after the *Deepwater Horizon* oil rig exploded on 20 April 2010, the American Academy of Pediatrics (AAP) worked with the Pediatric Environmental Health Specialty Units (PEHSU)^1^ to develop fact sheets for clinicians and parents that pulled together the best available information on protecting children against oil exposures.^2^ The recommendations, which have been endorsed by the American College of Medical Toxicology and the American Academy of Clinical Toxicology, cover a wide range of measures addressing air, water, beach, and food issues, and they can be applied in any oil-exposure situation.^3^

The recommendations are only professional best estimates based on the limited information about health effects of oil spills on adults, acknowledges Katherine Kirkland, executive director of the Association of Occupational and Environmental Clinics, which runs PEHSU. “There really isn’t any research done on children,” she says. The fact sheets will be updated as more information becomes available, says Scott Needle, a Florida pediatrician and member of the AAP Disaster Preparedness Advisory Council.

Along with physical health concerns, the fact sheets address psychological problems that may develop in children because of worries about their own health or that of their parents. Needle says it’s essential for parents to try to head off problems by talking with their children and to reassure them that people are working to clean up the oil and keep this from happening again.

“We are also concerned about the potential long-term community effects, particularly economic and psychological, and especially affecting areas that have already been battered by severe hurricanes and other stressors in recent years,” Needle says. “The majority of our experience in long-term effects comes from studying the *Exxon Valdez* spill in Alaska, and affected communities there are still dealing with the mental health effects.” Even if children do not exhibit health symptoms directly, he says, their overall health and sense of well-being may continue to be impacted on a long-term basis.

John Lanza, director of the Escambia County Health Department in Pensacola, Florida, had not heard of the PEHSU guidelines,^4^ but he says his county adopted similar recommendations based on research conducted by a consortium of Florida panhandle counties after the spill began. The county also conducted an extensive community education effort that parents and children seem to have heeded.

The number of acute health problems reported so far has been minimal.^5^ Data on longer-term effects on children may be hard to come by, however—of the four Gulf states tracking self-reported spill-related health complaints, only Louisiana is breaking out exposure and health effect data by age,^5^ and no definitive plans for researching health effects on children have surfaced. Moreover, as recently as mid-September new sightings of oil were being reported for several Louisiana beaches, marshes, and offshore areas,^6^ and there are widespread concerns that oil will continue to resurface in still other areas when storms pass through. It’s extremely difficult to predict how soon after any given storm that it will be safe to visit shoreline areas, says Stephen West, a Coast Guard spokesman working with the Unified Area Command.
